# Aromadendrin Protects Neuronal Cells from Methamphetamine-Induced Neurotoxicity by Regulating Endoplasmic Reticulum Stress and PI3K/Akt/mTOR Signaling Pathway

**DOI:** 10.3390/ijms22052274

**Published:** 2021-02-25

**Authors:** Hyun-Su Lee, Eun-Nam Kim, Gil-Saeng Jeong

**Affiliations:** College of Pharmacy, Keimyung University, Daegu 42601, Korea; hyunsu.lee@kmu.ac.kr (H.-S.L.); enkimpharm@gmail.com (E.-N.K.)

**Keywords:** METH, aromadendrin, neurotoxicity, SH-SY5y cells, protection, mTOR signaling

## Abstract

Methamphetamine (METH) is a highly addictive drug that induces irreversible damage to neuronal cells and pathological malfunction in the brain. Aromadendrin, isolated from the flowers of *Chionanthus retusus*, has been shown to have anti-inflammatory or anti-tumor activity. Nevertheless, it has been reported that METH exacerbates neurotoxicity by inducing endoplasmic reticulum (ER) stress via the phosphoinositide 3-kinase/Akt/mammalian target of rapamycin (PI3K/Akt/mTOR) pathway in neuronal cells. There is little evidence that aromadendrin protects cells from neurotoxicity induced by METH. In this study, we found that aromadendrin partially suppressed the METH-induced cell death in SH-SY5y cells without causing cytotoxicity. Aromadendrin regulated METH-induced ER stress by preserving the phosphorylation of the PI3K/Akt/mTOR signaling pathway in METH-exposed SH-SY5y cells. In addition, aromadendrin mitigated METH-induced autophagic and the apoptotic pathways in METH-exposed SH-SY5y cells. Mechanistic studies revealed that pre-treatment with aromadendrin restored the expression of anti-apoptotic proteins in METH-exposed conditions. The inhibitor assay confirmed that aromadendrin-mediated restoration of mTOR phosphorylation protected cells from autophagy and apoptosis in METH-exposed cells. Therefore, these findings suggest that aromadendrin relatively has a protective effect on SH-SY5y cells against autophagy and apoptosis induced by METH via regulation of ER stress and the PI3K/Akt/mTOR signaling pathway.

## 1. Introduction

Methamphetamine (METH) is a noxious phycostimulant that causes severe addiction to patients. Although several studies have reported the toxic effect of METH on human health, there are reports of a steady increase in METH-addicted patients [1,2]. In particular, accumulating investigations through cellular or animal experiments have demonstrated that METH induces neuronal toxicity, including autophagy and apoptosis in the brain [3]. METH-induced neurotoxic effects have been studied in relation to neurodegenerative diseases, including Alzheimer’s disease and Parkinson’s disease [4,5].

Autophagy is tightly regulated but a conserved mechanism for the recycling of degraded cellular resources [6]. Apoptosis is a vital process for maintaining cell homeostasis that is modulated by the balance between pro-apoptotic and anti-apoptotic proteins [7]. Recent reports have revealed that METH administration induces endoplasmic reticulum (ER) stress in neuronal cells [8,9]. ER stress induced by METH promotes the expression of CCAAT/enhancer-binding protein beta (C/EBPβ) and C/EBPβ homologous protein (CHOP). These molecular alterations lead to the autophagic pathway to enhance the expression of Beclin1 and microtubule-associated protein 1A/1B-light chain 3 (LC3) or apoptotic pathways to modulate the expression of the caspase family proteins via the PI3K/Akt/mTOR signaling pathway [10,11]. Nonetheless, ER stress induced by METH administration to neuronal cells causes uncontrolled cell death, little is known about whether bioactive molecules from natural products attenuate ER stress under METH exposure.

**Figure 1:**
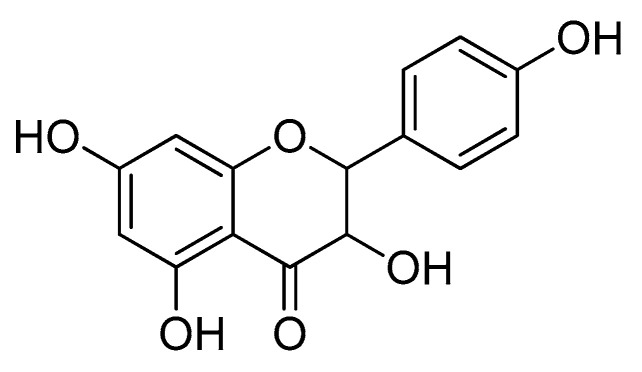
The chemical structure of aromadendrin.

Aromadendrin (Figure 1) is a bioactive flavonoid isolated from *Pinus sibirica*, *Afzelia bella*, and *Chioanathus retusus* [12,13,14]. It has been reported that aromadendrin has radical scavenging activity, anti-tumor, and anti-inflammatory activities [14,15,16]. Aromadendrin has been also evaluated that it possesses an anti-diabetic effect by upregulating insulin-mediated glucose uptake and by preserving insulin-stimulated Akt signaling [17]. A recent report has demonstrated the immunomodulatory effect of aromadendrin on T cell activation that pre-treatment with aromadendrin blocks calcium influx and nuclear factor of activated T-cells (NFAT) activity in T cell receptor mediated stimulation [18]. Anti-oxidative effect of aromadendrin was also elucidated through nuclear factor erythroid 2-related factor 2 (Nrf2)/heme oxygenase-1 (HO-1) pathway [18]. Although recovery of the Akt pathway is critical for the protective effect from METH toxicity [19], little is known whether aromadendrin shows a neuroprotective activity against METH-induced cytotoxicity. In the present study, we found that aromadendrin preserves SH-SY5y neuronal cells from METH-induced cytotoxicity by modulating ER stress and enhancing the PI3K/Akt/mTOR signaling pathway.

## 2. Results

### 2.1. Aromadendrin Does Not Lead to Neuronal Toxicity in SH-SY5y Cells

**Figure 2:**
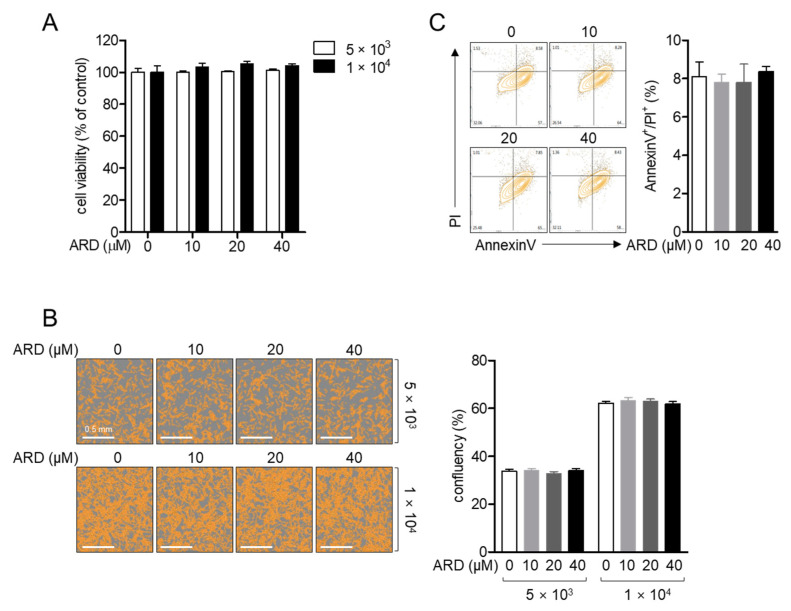
(**A**,**B**) SH-SY5y cells (5 × 10^3^/well or 1 × 10^4^/well, 96-well plate) were seeded and treated with the indicated concentration (0 to 40 μM) of aromadendrin for 24 h. Cell viability was measured by methyl thiazol tetrazolium (MTT) assay (A), and confluency was determined by IncuCyte imaging system (**B**). (**C**) Apoptotic cell population was evaluated by AnnexinV/propidium iodide (PI) apoptosis assay in SH-SY5y cells treated with the indicated concentration (0 to 40 μM) of aromadendrin for 24 h. White bar indicates 0.5 mm. The mean value of three experiments ± SEM is presented.

Previous reports have described that aromadendrin does not exert cytotoxic effects [18]. To confirm whether treatment with aromadendrin is cytotoxic to SH-SY5y neuronal cells, cell viability was assessed by methyl thiazol tetrazolium (MTT) assay with two different cell numbers per well. Figure 2A shows that treatment with up to 40 μM aromadendrin was not associated with cytotoxicity in SH-SY5y cells. DIC images also showed that aromadendrin did not affect cell confluency in the two different cell number conditions after 24 h of treatment (Figure 2B). To understand whether treatment with aromadendrin leads to the apoptotic pathway in SH-SY5y cells, AnnexinV/propidium Iodide (PI) apoptosis assay was performed. The population of cells undergoing apoptosis was independent of the concentration of aromadendrin used for treatment (Figure 2C). These results suggest that treatment with up to 40 μM aromadendrin is irrelevant to the cytotoxicity of SH-SY5y neuronal cells.

### 2.2. Aromadendrin Suppresses METH-Induced Cell Death in SH-SY5y Cells

**Figure 3:**
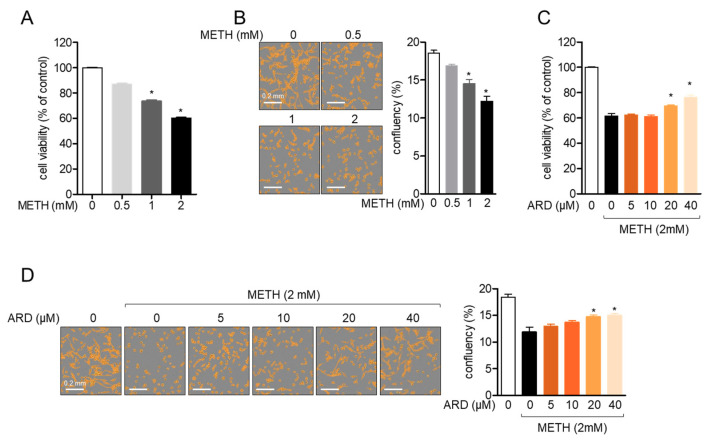
(**A**,**B**) SH-SY5y cells (5 × 10^3^/well, 96-well plate) were exposed to the indicated concentration (0 to 2 mM) of METH for 24 h. Viability was measured by MTT assay (**A**), and confluency was assessed by IncuCyte imaging system (**B**). (**C**,**D**) Pre-treated SH-SY5y cells (5 × 10^3^/well, 96-well plate) with the indicated concentration (0 to 40 μM) of aromadendrin for 1 h were exposed to 2 mM methamphetamine (METH) for 24 h. Cell viability was measured by MTT assay (**C**), and confluency was measured by IncuCyte imaging system (**D**). White bar indicates 0.2 mm. The mean value of three experiments ± SEM is presented. * *p* < 0.05 between mock-treated cells (**A**,**B**) or METH-treated cells (**C**,**D**).

It has been reported that treatment with METH induces cytotoxicity in several cells [20,21]. To confirm whether METH leads to cellular death in SH-SY5y cells, cell viability was measured after treatment with varying doses of METH for 24 h. Figure 3A reveals that viability was significantly reduced by METH in a dose-dependent manner (degree of freedom (*df*)= 11, *p* < 0.05 versus mock-treated). Confluency was also assessed to determine whether treatment with METH affects cell proliferation. Consistently reduced confluency was observed after treatment with METH (Figure 3B, *df* = 11, *p* < 0.05 versus mock-treated). To evaluate whether pre-treatment with aromadendrin protects neuronal cells from METH-induced cytotoxicity, cell viability was assessed by MTT assay in SH-SY5y cells pre-treated with aromadendrin for 1 h and exposed to 2 mM METH for 24 h. Figure 3C shows that pre-treatment with 20 μM aromadendrin relatively increased the viability of SH-SY5y cells exposed to 2 mM METH (*df* = 17, *p* < 0.05 versus METH-treated). In addition, the increase in the viability of the pre-treated cells was in a dose-dependent manner. Differential interference contrast (DIC) images obtained by the IncuCyte^®^ imaging system confirmed that the confluency of SH-SY5y neuronal cells was augmented by pre-treatment with aromadendrin in 2 mM METH-exposed condition (*df* = 11, *p* < 0.05 versus METH-treated). These results demonstrate that METH exposure induces neuronal cytotoxicity, but pre-treatment with aromadendrin suppresses the cytotoxicity induced by METH exposure.

### 2.3. Aromadendrin Regulates METH-Induced ER Stress in SH-SY5y Cells

**Figure 4:**
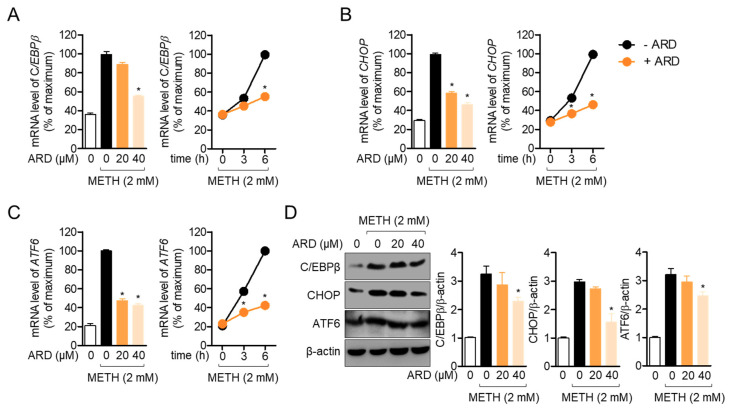
(**A**–**C**) SH-SY5y cells (1 × 10^5^/well, 6-well plate) were pre-treated with the indicated concentration (0 to 40 μM) of aromadendrin for 1 h and exposed to 2 mM METH for 6 h (left panel) or the indicated time (right panel). After collection, cells were lysed for real-time quantitative PCR analysis. The expression levels of indicated genes were determined and normalized with the level of *GAPDH*. (**D**) SH-SY5y cells (1 × 10^5^/well, 6-well plate) were pre-treated with the indicated concentration (0 to 40 μM) of aromadendrin for 1 h and treated with 2 mM METH for 24 h. Cells were harvested for Western blot analysis and indicated proteins were detected. The levels of indicated proteins were normalized with the level of β-actin. The mean value of three experiments ± SEM is presented. * *p* < 0.05 between METH-treated cells.

It has been shown that exposure to METH induces ER stress, which leads to neuronal cell death. To understand the underlying mechanism of the protective role of aromadendrin against METH-induced neuronal cytotoxicity, we first checked whether pre-treatment with aromadendrin affects METH-induced ER stress. The mRNA levels of *C/EBPβ* and *CHOP* were determined as markers of ER stress [10,11]. As shown in Figure 4A, the mRNA level of *C/EBPβ* was decreased by pre-treatment with aromadendrin in a time- (*df* = 11, *p* < 0.05 versus METH-treated) and dose-dependent manner (*df* = 17, *p* < 0.05 versus mock-treated). Figure 4B also reveals that pre-treatment with aromadendrin partially reduced the mRNA level of *CHOP* in METH-exposed neuronal cells (*df* = 11, *p* < 0.05 versus METH-treated and *df* = 17, *p* < 0.05 versus mock-treated, respectively). Suppressed mRNA level of activating transcription factor 6 (*ATF6)* was evaluated in METH-exposed cells pre-treated with aromadendrin (Figure 4C, *df* = 11, *p* < 0.05 versus METH-treated and *df* = 17, *p* < 0.05 versus mock-treated, respectively). Western blot analysis confirmed that pre-treatment with aromadendrin decreased the expression of ER stress markers at the protein level (Figure 4D, *df* = 11, *p* < 0.05 versus METH-treated). These data suggest that METH exposure leads to ER stress in neuronal cells, but pre-treatment with aromadendrin moderately attenuates ER stress induced by METH exposure.

### 2.4. Aromadendrin Preserves the Phosphorylation of PI3K/Akt/mTOR Signaling Pathway in METH-Exposed SH-SY5y Cells

**Figure 5:**
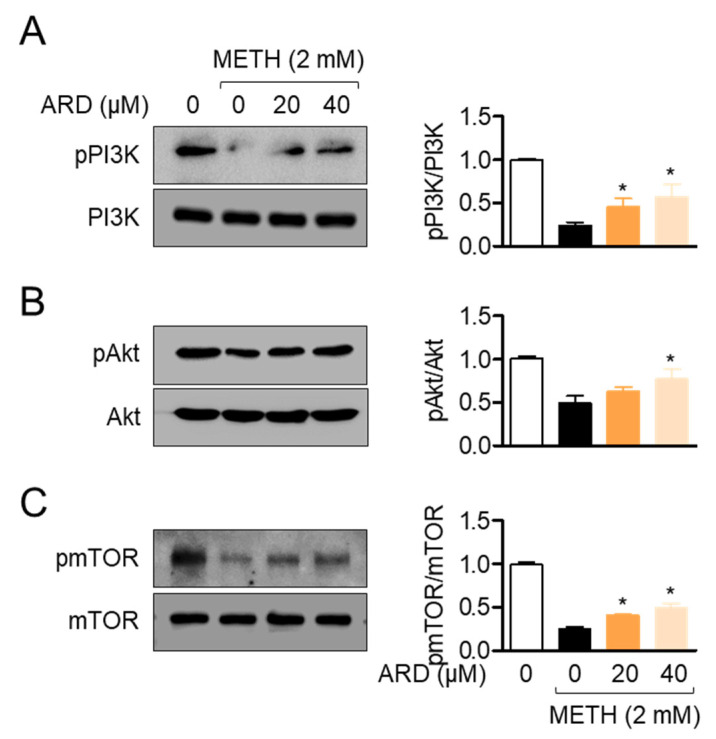
(**A**–**C**) SH-SY5y cells (1 × 10^5^/well, 6-well plate) were pre-treated with the indicated concentration (0 to 40 μM) aromadendrin for 1 h and exposed to 2 mM METH for 30 min. Harvested cells were lysed for Western blotting analysis to detect the phosphorylation level of PI3K (**A**), Akt (**B**), and mTOR (**C**). The phosphorylation levels of indicated proteins were normalized with the level of total proteins indicated. The mean value of three experiments ± SEM is presented. * *p* < 0.05 between METH-treated cells.

The PI3K/Akt/mTOR pathway has been established to play a central role in cell survival under ER stress conditions [22]. Since several studies have demonstrated that METH exposure leads to cytotoxicity by inhibiting the phosphorylation of PI3K/Akt/mTOR [23,24], we explored whether pre-treatment with aromadendrin is associated with the phosphorylation of PI3K/Akt/mTOR in METH-exposed neuronal cells. Figure 5A shows that the phosphorylation level of PI3K is reduced by METH exposure, but pre-treatment with aromadendrin relatively re-elevates the phosphorylation level of PI3K in SH-SY5y cells (*df* = 11, *p* < 0.05 versus METH-treated). Downstream signaling pathways of PI3K, including Akt (Figure 5B, *df* = 11, *p* < 0.05 versus METH-treated) and mTOR (Figure 5C), were also preserved by pre-treatment with aromadendrin in METH-exposed conditions (*df* = 11, *p* < 0.05 versus METH-treated). These data demonstrate that aromadendrin is partially involved in cell survival by restoring the phosphorylation of the PI3K/Akt/mTOR signaling pathway in METH-exposed neuronal cells.

### 2.5. Aromadendrin Mitigates the METH-Induced Autophagy in Neuronal Cells

**Figure 6:**
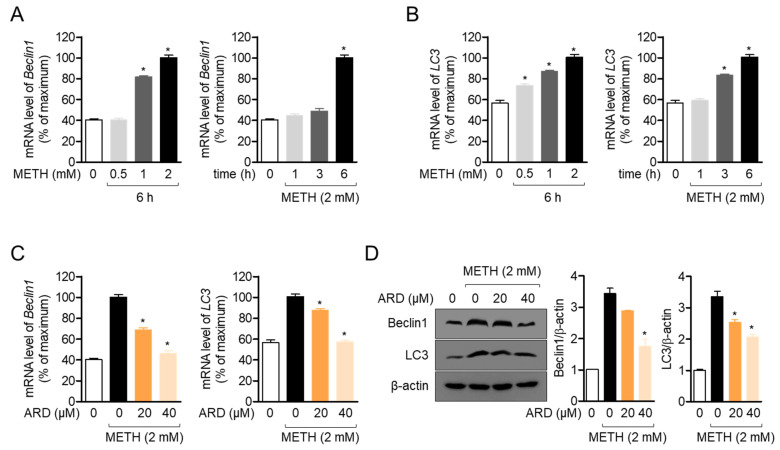
(**A**,**B**) SH-SY5y cells (1 × 10^5^/well, 6-well plate) were exposed to the indicated concentration (0 to 2 mM) of METH for 6 h or 2 mM of METH for the indicated time (0 to 6 h). After incubation, cells were collected for real-time quantitative (PCR analysis. The mRNA levels of *Beclin1* (**A**) and *LC3* (**B**) were detected and normalized with the level of *GAPDH*. (**C**) SH-SY5y cells (1 × 10^5^/well, 6-well plate) were pre-treated with the indicated concentration of aromadendrin (0 to 40 μM) for 1 h and exposed to 2 mM METH for 6 h. After incubation, cells were harvested and mRNA levels of *Beclin1* and *LC3* were determined by real-time quantitative PCR analysis. (**D**) SH-SY5y cells (1 × 10^5^/well, 6-well plate) were pre-treated with the indicated concentration (0 to 40 μM) of aromadendrin for 1 h and exposed to 2 mM METH for 24 h. Cells were collected for Western blot analysis and indicated proteins were detected. The levels of indicated proteins were normalized with the level of β-actin. The mean value of three experiments ± SEM is presented. * *p* < 0.05 between mock-treated cells (**A**,**B**) or METH-treated cells (**C**,**D**).

It has been shown that METH-induced neurocytotoxicity includes autophagy, which is one of the main manifestations in reduced phosphorylation of PI3K/Akt/mTOR pathway [10]. We showed that the mRNA level of *Beclin1*, a marker of the autophagic pathway, is elevated by METH exposure in a dose- and time-dependent manner (Figure 6A, *df* = 11, *p* < 0.05 versus mock-treated). Figure 6B shows that exposure to METH promotes the mRNA level of *LC3*, which plays a critical role in autophagy induction in a dose- and time-dependent manner (*df* = 11, *p* < 0.05 versus mock-treated). Since restored phosphorylation of the PI3K/Akt/mTOR pathway by pre-treatment with aromadendrin in METH-exposed conditions was checked, we further investigated whether pre-treatment with aromadendrin blocks the autophagic pathway induced by METH exposure. We found that elevated mRNA levels of *Beclin1* and *LC3* after METH exposure were moderately blocked by pre-treatment with aromadendrin in SH-SY5y cells (Figure 6C, *df* = 11, *p* < 0.05 versus METH-treated). A suppressive effect of aromadendrin on METH-induced autophagy was confirmed at the protein level (Figure 6D, *df* = 11, *p* < 0.05 versus METH-treated). These data suggest that exposure to METH induces the autophagic pathway in SH-SY5y cells, but pre-treatment with aromadendrin relatively mitigates the autophagy induced by METH exposure.

### 2.6. Pre-Treatment with Aromadendrin Has an Anti-Apoptotic Effect on METH-Exposed SH-SY5y Cells

**Figure 7:**
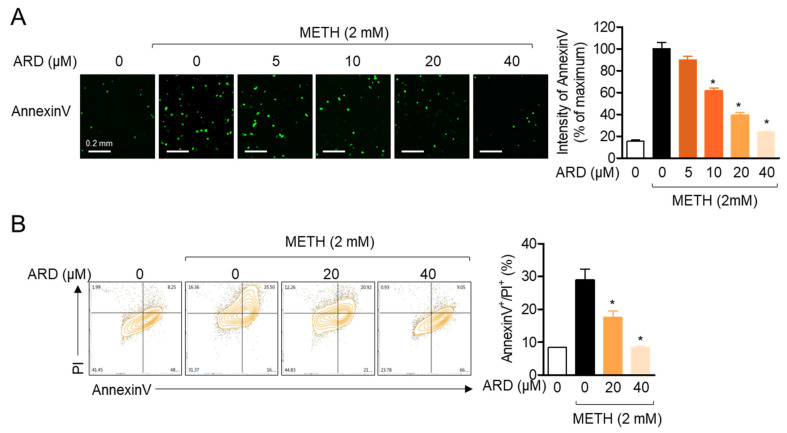
(**A**) SH-SY5y cells (5 × 10^3^/well, 96-well plate) stained with 1 × AnnexinV staining reagent were pre-treated with the indicated concentration (0 to 40 μM) of aromadendrin for 1 h and exposed to 2mM METH for 24 h. AnnexinV was detected by IncuCyte^®^ imaging system, and the intensity of AnnexinV was calculated. (**B**) SH-SY5y cells were pre-treated with the indicated concentration (0 to 40 μM) of aromadendrin for 1 h and exposed to 2 mM METH for 24 h. After collection, AnnexinV/PI apoptosis assay was performed by flow cytometry, and AnnexinV/PI-positive cells were presented in a bar graph. White bar indicates 0.2 mm. The mean value of three experiments ± SEM is presented. * *p* < 0.05 between METH-treated cells.

The other cytotoxic pathway induced by the suppression of the PI3K/Akt/mTOR survival pathway by METH is the apoptotic pathway. It has been widely reported that METH leads to the apoptotic pathway in neuronal cells [11,20]. To explore whether pre-treatment with aromadendrin has an anti-apoptotic effect against METH exposure, the intensity of AnnexinV was assessed in SH-SY5y cells using the IncuCyte^®^ imaging system. As shown in Figure 7A, exposure to 2mM METH augmented the intensity of AnnexinV in SH-SY5y cells, but pre-treatment with aromadendrin suppressed this effect in a dose-dependent manner (*df* = 17, *p* < 0.05 versus METH-treated). Results from the AnnexinV/PI apoptosis assay also showed that pre-treatment with aromadendrin downregulates the population of AnnexinV/PI-positive cells (Figure 7B, *df* = 11, *p* < 0.05 versus METH-treated). These data demonstrate that pre-treatment with aromadendrin partially restricts METH-induced apoptosis in SH-SY5y neuronal cells.

### 2.7. Pre-Treatment with Aromadendrin Modulates the Expression of Apoptosis-Related Proteins in METH-Exposed Condition

**Figure 8:**
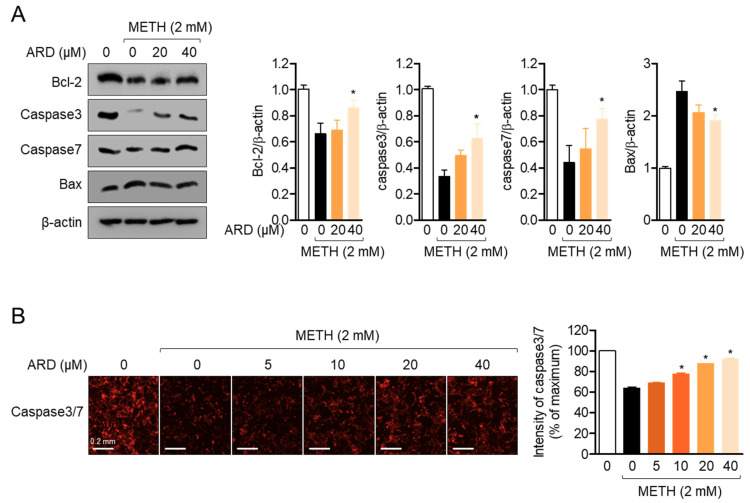
(**A**) SH-SY5y cells (1 × 10^5^/well, 6-well plate) were pre-treated with the indicated concentration (0 to 40 μM) of aromadendrin for 1 h and exposed to 2 mM METH for 24 h. After harvested, cells were lysed for Western blotting analysis. The indicated proteins were detected and normalized with the level of β-actin. (**B**) Stained SH-SY5y cells (5 × 10^3^/well, 96-well plate) with 1 × Caspase3/7 staining reagent were pre-treated with the indicated concentration (0 to 40 μM) of aromadendrin for 1 h and exposed to 2 mM METH for 24 h. The expression of Caspase3/7 was detected, and the intensity of Caspase3/7 was measured using the IncuCyte^®^ imaging system. White bar indicates 0.2 mm. The mean value of three experiments ± SEM is presented. * *p* < 0.05 between METH-treated cells.

To investigate whether pre-treatment with aromadendrin affects the expression of apoptosis-related proteins in METH exposed conditions, the expression levels of the caspase family of proteins and Bax were determined by Western blot analysis. Figure 8A shows that METH exposure in SH-SY5y cells downregulated the expression of Bcl-2, Caspase 3, and Caspase7, which are anti-apoptotic proteins, but increased the expression of Bax, which promotes the apoptotic pathway in SH-SY5y cells. However, pre-treatment with aromadendrin slightly elevated the expression of Bcl-2, Caspase3, and Caspase7 but reduced the expression of Bax in METH-exposed SH-SY5y cells (*df* = 11, *p* < 0.05 versus METH-treated). The fluorescent detection of Caspase3/7 by the IncuCyte^®^ imaging system also confirmed that pre-treatment with aromadendrin preserves the reduction of Caspase3/7 intensity by METH exposure in a dose-dependent manner (*df* = 17, *p* < 0.05 versus METH-treated). These data suggest that pre-treatment with aromadendrin moderately restores the expression of anti-apoptotic proteins but suppresses the expression of pro-apoptotic proteins under conditions of METH exposure.

### 2.8. Restored mTOR Phosphorylation by Aromadendrin Protects Cells from Autophagy and Apoptosis

**Figure 9:**
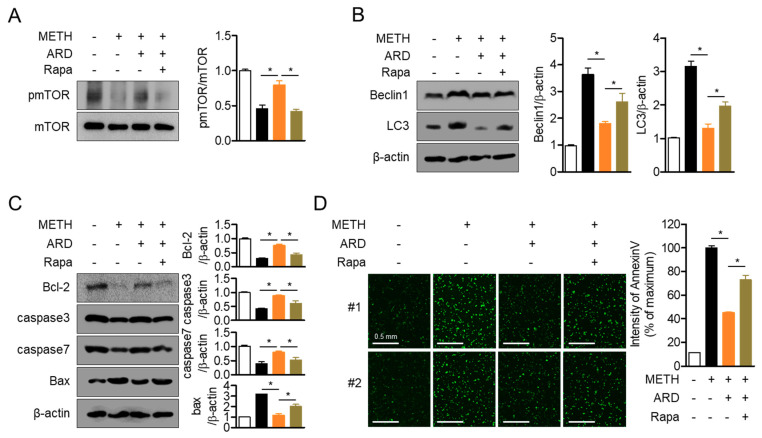
(**A**) SH-SY5y cells (1 × 10^5^/well, 6-well plate) were pre-treated with 50 μM rapamycin for 1 h and then 40 μM of aromadendrin for 1 h. Cells exposed to 2 mM METH for 30 min were harvested for Western blotting analysis. The phosphorylation level of mTOR was detected and normalized with the total level of mTOR. (**B**,**C**) SH-SY5y cells (1 × 10^5^/well, 6-well plate) were pre-treated with 50 μM rapamycin for 1 h and then 40 μM of aromadendrin for 1 h. Cells were incubated with 2 mM METH for 24 h and collected for Western blotting analysis. The level of indicated proteins was measured by Western blotting analysis and normalized with the level of β-actin. (**D**) SH-SY5y cells (5 × 10^3^/well, 96-well plate) stained with 1 × AnnexinV staining reagent were pre-treated with 50 μM rapamycin for 1 h and then pre-treated with 40 μM aromadendrin for 1 h. Cells were exposed to 2 mM METH for 24 h, and the expression of AnnexinV was detected using the IncuCyte^®^ imaging system. The intensity of AnnexinV was calculated by IncuCyte^®^ imaging system software. White bar indicates 0.5 mm. The mean value of three experiments ± SEM is presented. * *p* < 0.05 between the two indicated groups.

The mTOR signaling pathway plays a critical role in modulating the fate of cells in autophagic or apoptotic pathways in METH exposed conditions. To explore whether pre-treatment with aromadendrin has a protective effect against METH-induced neurotoxicity via the mTOR signaling pathway, an inhibitor assay was performed using rapamycin, a potent inhibitor of mTOR phosphorylation. Figure 9A shows that the promoting effect of aromadendrin on mTOR phosphorylation is regulated by pre-treatment with rapamycin in METH-exposed conditions (*df* = 11, *p* < 0.05 between two indicated group). It was confirmed that the anti-autophagic effect of aromadendrin in METH-exposed conditions was removed by pre-treatment with rapamycin (Figure 9B, *df* = 11, *p* < 0.05 between two indicated group). In addition, Figure 9C also confirms that pre-treatment with rapamycin eliminated the anti-apoptotic activity of aromadendrin in METH-exposed conditions (*df* = 11, *p* < 0.05 between two indicated group). The fluorescence of AnnexinV detected by the IncuCyte^®^ imaging system also demonstrated that pre-treatment with rapamycin relatively inhibited the protective effect of aromadendrin in METH-exposed conditions (Figure 9D, *df* = 11, *p* < 0.05 between two indicated group). These data indicate that restored mTOR phosphorylation by aromadendrin preserves neuronal cells from autophagy and apoptosis induced by METH exposure.

## 3. Discussion

In the present study, we found an ameliorating effect of aromadendrin isolated from *C. retusus* flowers on the toxicity induced by METH exposure in neuronal cells. Quantitative PCR and Western blot analysis demonstrated that pre-treatment with aromadendrin reduced the ER stress induced by METH exposure. Reduction of ER stress by aromadendrin pre-treatment preserved the phosphorylation of PI3K/Akt/mTOR pathway and showed anti-autophagic and anti-apoptotic effects on SH-SY5y neuronal cells under conditions of METH exposure. The inhibitor assay confirmed that restored mTOR phosphorylation by aromadendrin ameliorates to undergo autophagy and the apoptosis pathway in METH-exposed conditions.

Several flavonoids, a member of aromadendrin, have been investigated to alleviate methamphetamine-induced toxicity in vitro and in vivo recently. Epigallocatechin gallate found in green tea has been demonstrated to attenuate METH-induced striatal dopamine terminal toxicity through modulation of oxidative stress in brain tissue [25]. Luteolin, widely used in traditional Chinese medicine, has been exhibited that oral administration of luteolin effectively protects rats from METH-induced neurotoxicity including apoptosis and autophagy via PI3K/Akt pathway [26]. A recent report has elucidated that pre-treatment with epicatechin has a neuroprotective effect on METH-induced cell death through suppression of ER stress [27]. Since various flavonoids including aromadendrin have been studied to show a potent neuroprotective effect against METH-induced cytotoxicity, the strategy of using bioactive flavonoids in developing disease treatments caused by METH abuse is highly promising.

Various studies have demonstrated that METH induces ER stress, and this induction is highly involved in neurodegenerative diseases [28,29,30]. Several molecules have been investigated to play a critical role in the METH-mediated ER stress pathway. A recent publication has demonstrated the involvement of ATF6, inositol-requiring enzyme (IRE) and pancreatic ER elF2a kinase (PERK) located on the ER membrane in the uptake of METH into cells [31]. It has also been discovered that METH leads to ER stress through the ER signaling pathway including IRE, PERK and ATF6. Since most of neurodegenerative disorders are related to the accumulation of misfolded proteins that causes ER stress, alleviation of ER stress induced by METH exposure would be one of the potential pharmacological approach to overcome side effects of METH addiction. In the present study, we found that pre-treatment with aromadendrin inhibits the induction of C/EBPβ and CHOP by METH exposure in neuronal cells and results in the reduction of ER stress by METH (Figure 4). These data assume that pre-treatment with aromadendrin attenuates ER signaling pathway caused by METH exposure.

The PI3K/Akt/mTOR pathway is one of the most critical pathways that modulate numerous bioactivities, including cell survival, cell death, and cell growth. The phosphorylation cascades of the pathway are tightly regulated by several kinases and phosphatases in the presence of stimulants. It has been elucidated that METH treatment promotes the autophagic and apoptotic pathway by dephosphorylating the PI3K/Akt/mTOR pathway in various cells. Although the mechanism by which METH reduces the level of phosphorylation in PI3K/Akt/mTOR is still unclear, recent literature suggests that ER stress induced by METH exposure leads to dephosphorylation of the PI3K/Akt/mTOR pathway. Our current study demonstrated that pre-treatment with aromadendrin protects against the dephosphorylation of PI3K/Akt/mTOR induced by METH exposure by suppressing ER stress (Figure 4). However, pre-treatment with the mTOR inhibitor (rapamycin) significantly downregulated the protective effect of aromadendrin on autophagy and apoptosis induced by METH (Figure 9). These results suggest that the ameliorative effects of aromadendrin on autophagy and apoptosis are not only due to reduced ER stress, but also direct modulation of aromadendrin in PI3K/Akt/mTOR dephosphorylation. The mechanism underlying the involvement of aromadendrin in the phosphorylation of PI3K/Akt/mTOR should be investigated in a future study.

## 4. Materials and Methods

### 4.1. Cells

SH-SY5y human neuronal cells were purchased from the Korean Cell Line Bank (KCLB No. 22266, Seoul, Korea). The cells were identified with STR profiling, including D7S820, D13S317, FGA, TPOX, and TH01 by KCLB before distribution. The cells were cultured in DMEM medium (Welgene, Gyeongsan-si, Korea) supplemented with 10% fetal bovine serum (FBS), penicillin G (100 units/mL), l-glutamine (2 mM), and streptomycin (100 μg/mL) at 37 °C in a humidified incubator containing 5% CO_2_. Cells were maintained within passage #10 for all experiments.

### 4.2. Isolation of Aromadendrin from C. retusus Flowers

Aromadendrin (C_15_H_12_O_6_) was isolated from the flowers of *C. retusus*, as previously reported [14,32]. After the dried *C. retusus* flowers were extracted with MeOH, the MeOH extract was evaporated under reduced pressure, and the residue was suspended in H_2_O. The suspended MeOH extract was partitioned into EtOAc, n-BuOH, and H_2_O. The EtOAc fraction was separated by Sephadex LH-20 column chromatography under the elution conditions of EtOAc-MeOH (15:15:1, 10:10:2) to obtain 10 fractions (Fr.1–10). Fr.3 was further separated to obtain five subfractions (Fr.3-1–Fr.3-5). Subfraction Fr.3-2 was purified with Sephadex LH-20 (MeOH) to obtain compound 1. The isolated Compound 1 was identified as aromadendrin as a result of NMR spectroscopy (JEOL JNM-ECA 500) analysis compared to published literature [14]. ^1^H-NMR spectra of aromadendrin was shown in the Figure S1.

Aromadendrin; ^1^H-NMR (500 MHz, DMSO-*d*_6_) δ: 4.54 (1*H*, brdd, *J*= 11.4, 3.8 Hz, H-3), 4.99 (1*H*, d, *J*= 11.4 Hz, H-2), 5.69 (1*H*, d, *J*= 2.2 Hz, H-6), 5.82 (1*H*, d, *J*= 2.2 Hz, H-8), 6.74 (2H, d, *J*= 8.8 Hz, H-3’, 5’), 7.26 (2*H*, d, *J*= 8.8 Hz, H-2’, 6’), 9.51 (1*H*, s, 7-OH), 11.88 (1*H*, s, 5-OH).

### 4.3. Reagents and Antibodies

MTT powder (1-(4,5-Dimethylthiazol-2-yl)-3,5-diphenylformazan), rapamycin, METH powder, TRIZOL reagent, and RIPA buffer were purchased from Sigma-Aldrich (St. Louis, MO, USA). Staining reagents for AnnexinV and Caspase3/7 were obtained from Essen Bio (Ann Arbor, MI, USA). AnnexinV/PI apoptosis assay kit was purchased from BD Biosciences (San Diego, CA, USA). Antibodies against C/EBPβ, PI3K, Akt, mTOR, phosphorylated PI3K, phosphorylated Akt, Beclin1, LC3, Caspase3, Caspase7, and Bax were obtained from Cell Signaling Technology (Danvers, MA, USA). Anti-CHOP, anti-phosphorylated mTOR, anti-Bcl2, and anti-β-actin antibodies were purchased from Santa Cruz Biotechnology (Dallas, TX, USA). Antibodies against ATF6 were obtained from Abcam (Cambridge, UK). SYBR Premix Ex Taq was purchased from Takara (Shiga, Japan). The RT PreMix kit was obtained from Enzynomics (Daejeon, Korea). ECL Western blotting detection reagents were purchased from Thermo Fisher Scientific (Waltham, MA, USA).

### 4.4. MTT Assay

MTT assay was performed to evaluate cytotoxicity as a reference [18]. Briefly, seeded SH-SY5y cells (5 × 10^3^/well or 1 × 10^4^/well, 96-well plate) were incubated in the indicated conditions for 24 h. The supernatants were removed, and cells were treated with 500 μg/mL of MTT for 1 h. Supernatants were discarded, and the generated formazan crystals were dissolved with 160 μL of DMSO. The plate was read to obtain the absorbance at 540 nm, and cell viability was calculated by comparing the reading with the absorbance of control cells (% of control). Mean values ± SEM were calculated from the data acquired from three independent experiments performed on separate days and presented as a bar graph. One-way ANOVA was used to determine significance (*p* value). * indicates differences between mock-treated considered significant at *p* < 0.05.

### 4.5. Assessment of Cell Confluency

Cell confluency was assessed as a reference [33]. Seeded SH-SY5y cells (5 × 10^3^/well or 1 × 10^4^/well, 96-well plate) were incubated in the indicated conditions for 24 h and were scanned to assess the cellular confluency using the IncuCyte^®^ imaging system. Cells on the plate were marked in orange after recognition as cells in the software. Confluency was automatically determined according to the area of orange color and presented as a bar graph.

### 4.6. AnnexinV/PI Apoptosis Assay by Flow Cytometry

Apoptosis using AnnexinV and PI staining was performed as a reference [20]. SH-SY5y cells undergoing the apoptotic pathway were determined by double staining using AnnexinV and PI. After treatment of SH-SY5y cells with the indicated conditions for 24 h, the cells were suspended in 1X Trypsin-EDTA buffer and resuspended in 100 μL of 1× binding buffer containing AnnexinV (20 μg/mL) and PI (1 μg/mL) for 30 min on ice. Stained cells were acquired by flow cytometry (BD FACSVerse, BD Biosciences, San Diego, CA, USA), and the population of Annexin V^+^/PI^+^ cells is shown in the bar graph with plots. Mean values ± SEM were calculated from the data acquired from three independent experiments performed on separate days and presented as a bar graph. One-way ANOVA was used to determine significance (*p* value). * indicates differences between indicated control in each figure legend, considered significant at *p* < 0.05.

### 4.7. Real-Time Quantitative PCR

Total RNA was isolated from harvested cells treated in the indicated conditions using TRIZOL reagent, and reverse transcription of the total RNA to cDNA was performed. Primers used for each gene were as follows (forward and reverse primers, respectively): human *C/EBPb*, 5′-AGA AGA CCG TGG ACA AGC ACA G-3′ and 5′-CTC CAG GAC CTT GTG CTG CGT-3′; human *CHOP*, 5′-TGC TTC TCT GGC TTG GCT GAC-3′ and 5′-CCG TTT CCT GGT TCT CCC TTG G-3′, human *ATF6*, 5′-GCT TTA CAT TCC TCC ACC TCC TTG-3′ and 5′-ATT TGA GCC CTG TTC CAG AGC AC-3′, human *BECLIN1*, 5′-AGC TGC CGT TAT ACT GTT CT-3′ and 5′-TGT GTC TTC AAT CTT GCC TT-3′, human *LC3*, 5′-GAG AAG CAG CTT CCT GTT CTG G-3′ and 5′-GTG TCC GTT CAC CAA CAG GAA G-3′, human *GAPDH*, 5′-CGG AGT CAA CGG ATT TGG TCG TAT-3′ and 5′-AGC CTT CTC CAT GGT GGT GAA GAC-3′. For quantitative PCR analysis, amplification was performed in a DNA Engine Opticon 1 continuous fluorescence detection system (MJ Research, Waltham, MA, USA) using SYBR Premix Ex Taq. The total reaction volume was 10 μL containing 0.1 μg of cDNA, and each PCR reaction was performed using the following conditions: 95 °C for 30 s, 60 °C for 20 s, and plate read for 40 cycles followed by 7 min of extension at 70°C. Melting curve analysis was performed to characterize the dsDNA product by slowly increasing the temperature (0.2 °C/s) from 60 °C to 97 °C with fluorescence data collected at 0.2°C intervals. mRNA levels of genes were normalized with mRNA levels of *GAPDH* and presented as % of maximum. The % of maximum was calculated using the following equation: % of maximum = 2^−ΔΔCT^ × 100, where ΔΔCT = (CT_target_ − CT_GAPDH_) at maximum−(CT_target_ − CT_GAPDH_). Mean values ± SEM were calculated from the data acquired from three independent experiments performed on separate days and presented as a bar graph. One-way ANOVA was used to determine significance (*p* value). * indicates differences between indicated control in each figure legend, considered significant at *p* < 0.05.

### 4.8. Western Blot Analysis

Western blot analysis was performed as a reference [20]. SH-SY5y cells incubated in the indicated conditions were collected for lysis in RIPA buffer with 1× phosphatase inhibitor on ice for 15 min. Lysates were then centrifuged at 13,500× rpm at 4 °C for 25 min, and 30 μg of the lysate was loaded for separation on 6–15% SDS–PAGE gels. Proteins were transferred onto PVDF membranes, and membranes were blocked with 5% skim milk in 0.1% TBS-T for 1 h. After blocking, membranes were incubated with the indicated primary antibodies in 3% skim milk overnight (1:1000 ratio). Excess primary antibodies were discarded by washing the membrane three times with TBS-T and incubated with 0.1 μg/mL peroxidase-labeled secondary antibodies (against rabbit or mouse) for 2 h. After three washes with 0.1% TBS-T, bands were detected with ECL Western blot detection reagents with an ImageQuant LAS 4000 (GE Healthcare, Chicago, IL, USA). Mean values ± SEM were calculated from the data acquired from three independent experiments performed on separate days and presented as a bar graph. One-way ANOVA was used to determine significance (*p* value). * indicates differences between indicated control in each figure legend, considered significant at *p* < 0.05.

### 4.9. Determination of AnnexinV and Caspase3/7 Expression by IncuCyte^®^ Imaging System

Evaluation of AnnexinV and Caspase3/7 expression by IncuCyte^®^ imaging system was performed as a reference [18]. Pre-stained SH-SY5y cells with 1× AnnexinV or Caspase3/7 staining reagents were incubated in the indicated conditions, and the intensity of AnnexinV or Caspase3/7 was determined by scanning the cells using IncuCyte^®^ imaging system. The % of maximum was calculated for presentation in a bar graph. Mean values ± SEM were calculated from the data acquired from three independent experiments performed on separate days and presented as a bar graph. One-way ANOVA was used to determine significance (*p* value). * indicates differences between indicated control in each figure legend, considered significant at *p* < 0.05.

## Data Availability

The data presented in this study are available on request from the corresponding author.

## Supplementary Materials

The following are available online at https://www.mdpi.com/1422-0067/22/5/2274/s1, Figure S1: ^1^H-NMR spectra of aromadendrin (500 MHz, DMSO-*d6*).

## Abbreviations

ARD	Aromadendrin
METH	Methamphetamine
mTOR	Mammalian target of rapamycin
PI	Propidium iodide
