# Supplementary material for: Aromadendrin Protects Neuronal Cells from Methamphetamine-Induced Neurotoxicity by Regulating Endoplasmic Reticulum Stress and PI3K/Akt/mTOR Signaling Pathway
Source: Int J Mol Sci. 2021 Feb 25;22(5):2274. doi: 10.3390/ijms22052274 (PMC7956189; doi:10.3390/ijms22052274)
Supplement: Supplementary file 1 [file ijms-22-02274-s001.pdf]

International Journal of
Molecular Sciences

MDPI

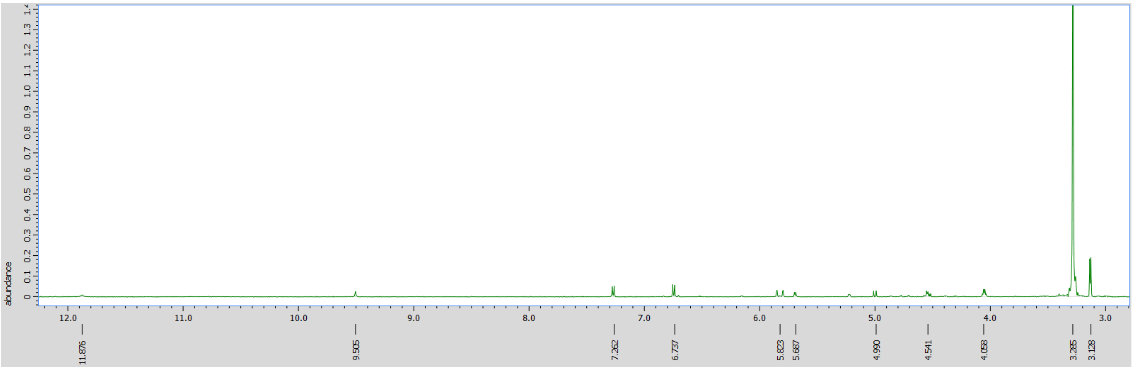

Figure S1. ^{1}H-NMR spectra of aromadendrin (500 MHz, DMSO-d_6). Aromadendrin ^{1}H-NMR (500 MHz, DMSO-d_6) δ : 4.54 (1*H*, brdd, *J*= 11.4, 3.8 Hz, H-3), 4.99 (1H, d, *J*= 11.4 Hz, H-2), 5.69 (1*H*, d, *J*= 2.2 Hz, H-6), 5.82 (1*H*, d, *J*= 2.2 Hz, H-8), 6.74 (2*H*, d, *J*= 8.8 Hz, H-3', 5'), 7.26 (2*H*, d, *J*= 8.8 Hz, H-2', 6'), 9.51 (1*H*, s, 7-OH), 11.88 (1*H*, s, 5-OH).
